# Reviewers for the *Journal of Insect Science* (January 2019–October 2019)

**DOI:** 10.1093/jisesa/iez115

**Published:** 2020-01-06

The Editor-in-Chief and the Subject Editors of the *Journal of Insect Science* thank the following scientists for their voluntary commitment of valuable professional time and expertise to peer reviewing manuscripts submitted for publication in our journal. The quality and scientific stature of the journal depends on the conscientious efforts of these individuals.

**Top reviewers (completed 3+ reviews)**

Athanassiou, Christos

Chaudhury, Muhammad

Evenden, Maya

Forbes, Andrew

Fourcade, Yoan

Lapointe, Stephen

Orlova-Bienkowskaja, Marina

Weintraub, Phyllis

Zalucki, Myron

**Reviewers**

Adelman, Zach

Adler, Lynn S.

Ahmed, Muhammad Z.

Allan, Sandra

Allen, Margaret

Alves, Tavvs

Amaral, Karina Dias

Ameye, Maarten

Ammar, El-Desouky

Antony, Binu

Araujo, Douglas

Armijos-Jaramillo, Vinicio

Armisén, David

Asplen, Mark

Atlihan, Remzi

Azhar, Badrul

Baishya, Bhavna

Bartels, David

Bastola, Anup

Battisti, Andrea

Ben-Shahar, Yehuda

Bendena, Bill

Berardi, Laura

Berkov, Amy

Boissot, Nathalie

Borges, Paulo

Boulton, Rebecca

Broughton, Sonya

Brunet, Johanne

Buchholz, Sascha

Burrows, Malcolm

Buser, Claudia

Candan, Selami

Cardoso, Hélia

Carvalho, Ana Paula

Cato, Aaron

Chappell, Thomas

Chen, Fajun

Chen, Hui

Chen, Maohua

Chen, Ming

Chimonyo, M.

Chitturi, Anitha

Christiaens, Olivier

Ciampor, Fedor

Cloonan, Kevin

Coquerel, Quentin

Cribb, Bronwen

Cristiano, Maykon

Cunningham, Paul

Da Lage, Jean-Luc

Dale, Adam

Daugherty, Matt

Davis, Jeffrey

de Beer, Chantel

De Marchi, Bruno

de Morais Tintino, Cicera Datiane

Dedos, Skarlatos

Deng, Arop

Dias, Naymã

Dindo, Maria Luisa

Dittmer, Neal

Djordjevic, Mirko

Dou, Xiaoyi

Economou, Leonidas

Ehlers, Ralf-Udo

Eigenbrode, Sanford

Ejigu, Ephrem A

Ellsworth, Peter

Etebari, Kayvan

Fausto, Anna Maria

Fellows, Christopher

Fereres, Alberto

Ferguson, Laura V.

Ferrante, Marco

Fiedler, Konrad

Finlayson, Ewan D.

Fónagy, Adrien

Forman, Martin

Fragoso, Fabiana

Franzén, Markus

Freitas, Alex

Friesen, Kevin

Galuppi, Roberta

Gavish-Regev, Efrat

George, Justin

Gerry, Alec

Giannoulis, Pascalis

Giray, Tugrul

Godfrey, Kris

Gonzalez, Ezequiel

Gottlieb-Dror, Yuval

Gratton, Claudio

Graystock, Peter

Greb-Markiewicz, Beata

Grimaldi, Annalisa

Grove, Tertia

Gui, FuRong

Güncan, Ali

Gunter, Nicole L.

Haelewaters, Danny

Hahn, Noel

Hancock, Geoffrey

Hanna, Catherine J. B.

Harrison, Kyle

Hassall, Christopher

Haye, Tim

Hazir, Selcuk

Head, Graham

Headrick, David

Hedstrom, Christopher

Hemberger, Jeremy

Hoelmer, Kim

Horigane, Mari

Hou, Qiuli

Huang, Fangneng

Hull, Joe

Husemann, Martin

Hutchison, William

Imbahale, Susan

Janssen, Dirk

Jesus Navarro, Maria

Jing, Shengli

Jinjie, Cui

Jones, Andrew

Joyce, Andrea

Jurat-Fuentes, Juan

Jurenka, Russell

Kainoh, Yooichi

Kapheim, Karen

Karimi, Javad

Kaunisto, Kari

Kawazu, Kei

Kennedy, George

Klein, Barrett

Kodandaramaiah, Ullasa

Kodrík, Dalibor

Kolliopoulou, Anna

Koto, Akiko

Kourimska, Lenka

Krell, Frank

Krempl, Corinna

Kwapich, Christina

Laskowski, Ryszard

Lau, Pierre

Leak, Stephen

Legg, James

Leite, Natália

Letsch, Harald

Li, Jianhong

Ling, Erjun

Liu, Chenxi

Liu, Feng

Liu, Tong-Xian

Liu, Wen

Lord, Nathan

Loza-Mejía, Marco A.

Lu, Zhiqiang

Lubin, Yael

Ludwick, Dalton

Ma, Weihua

Maebe, Kevin

Malysh, Julia M.

Manfredini, Fabio

Martin, Arnaud

Martínez-Ávila, Guillermo

Mayfield, Albert

McGregor, Rob

Meersman, Filip

Mendoza-Galvan, Arturo

Millar, Jocelyn G.

Missirlis, Fanis

Moadeli, Tahereh

Moisander, Pia H.

Morales-Ramos, Juan

Moran, Patrick

Mussury, RM

Neven, Lisa

Nicodemo, Daniel

Nooten, Sabine

O’Neal, Scott

Oderkirk, Nancy

Okada, Kensuke

Ono, Hajime

Opatovsky, Itai

Osbrink, Weste

Otaki, Joji

Owens, D

Pagabeleguem, Soumaila

Pascacio-Villafán, Carlos

Pasookhush, Phongthana

Paula, D. P.

Pedrini, Nicolas

Pelz-Stelinski, Kirsten

Peng, Yu

Pereira, Eliseu

Pérez-Staples, Diana

Peterson, Jessica

Pickett, Charles

Portilla, Maribel

Pozsgai, Gabor

Prado Maluta, Nathalie

Qu, Cheng

Quan, Fu-Shi

Rahman, Sarthok

Rajarapu, Swapna Priya

Reddy, B. Venugopal

Reisig, Dominic

Reynolds, Donald R.

Roberts, Phillip

Rodríguez-Leyva, Esteban

Rondoni, Gabriele

Rossini, Michele

Ruiu, Luca

Rull, Juan

Sabater-Muñoz, Beatriz

Sakka, Maria

Salminen, Juha-Pekka

Samuels, Richard

Sanders, Christopher J.

Santana, Paulo

Schal, Coby

Schmitt, Michael

Schneider, John

Schneider, Marielle Cristina

Schwartz, Howard

Segoli, Michal

Sen, Sandeep

Serrão, José Eduardo

Seshadri, Arathi

Shao, Yongqi

Sharakhova, Maria

Shelby, Kent

Shepherd, William

Shirotsuka, Kanako

Silva, Carlos

Simone-Finstrom, Mike

Siviter, Harry

Smith, Hugh

Snoddy, Edward

Song, Nan

Soulages, Jose L.

Sovik, Eirik

Sparks, Michael

Srinivasan, Rajagopalbabu

Stockton, Dara

Styrsky, John

Su, Jianya

Sundaram, Janarthanan

Svacha, Peter

Swale, Daniel

Szczepaniec, Adrianna

Tack, Ayco

Tait, Gabriella

Tammaru, Toomas

Tessnow, Ashely

Tognon, Roberta

Tomanovic, Zeljko

Tschinkel, Walter

Tsubota, Takuya

Van Dyck, Hans

Vargas, Héctor

Varricchio, Paola

Vasudeva, Ramakrishnan

Verheggen, François

Vilcinskas, Andreas

Vogel, Courtney

Vogel, Heiko

Vosteen, Ilka

Wada-Katsumata, Ayako

Wakie, Tewodros

Wang, Jianjun

Wang, Lei

Wang, Ran

Wang, Xiaowei

Wang, Yongmo

Way, M.

Wayadande, Astri

Wee, Suk-Ling

Welch, John J.

Wessely, Johannes

Wieczorek, Karina

Wiemers, Martin

Willett, Denis

Willmott, Keith

Wojcik, Victoria

Worthington, Amy M.

Wu, Hao

Wu, Shunfan

Xie, Wen

Xu, Jia-Ping

Yang, Chin-Cheng

Yao, Jianxiu

Yu, Xiao-Qiang

Zemanova, Miriam A.

Zhan, Yinpeng

Zhang, Jibin

Zhu, Junwei (Jerry)

Zhu, Li

Zhu, Yu-Cheng

